# Plasma YKL-40 and all-cause mortality in patients with chronic obstructive pulmonary disease

**DOI:** 10.1186/1471-2466-13-77

**Published:** 2013-12-30

**Authors:** Dennis B Holmgaard, Lone H Mygind, Ingrid L Titlestad, Hanne Madsen, Svend Stenvang Pedersen, Julia S Johansen, Court Pedersen

**Affiliations:** 1 Department of Infectious Diseases Q, Odense University Hospital, DK-5000 Odense C, Denmark; 2 Department of Respiratory Medicine J, Odense University Hospital, Odense, Denmark; 3 Department of Infectious Diseases, Aalborg Hospital, Aalborg, Denmark; 4 Departments of Medicine and Oncology, Herlev Hospital, Copenhagen University Hospital, University of Copenhagen, Copenhagen, Denmark

**Keywords:** COPD, Inflammation, Mortality, Prognosis, YKL-40

## Abstract

**Background:**

Chronic obstructive pulmonary disease (COPD) is hallmarked by inflammatory processes and a progressive decline of lung function. YKL-40 is a potential biomarker of inflammation and mortality in patients suffering from inflammatory lung disease, but its prognostic value in patients with COPD remains unknown. We investigated whether high plasma YKL-40 was associated with increased mortality in patients with moderate to very severe COPD.

**Methods:**

Four hundred and ninety-three patients with moderate to very severe COPD were followed prospectively for up to 10 years. Patients were divided into two groups according to plasma YKL-40: concentration higher than the 75^th^ percentile for age-matched healthy subjects (i.e. high levels) and normal levels. Outcome was overall survival (OS) and was evaluated in uni- and multivariate proportional hazards Cox regression analyses and adjusted for factors affecting mortality.

**Results:**

Median plasma YKL-40 was increased in patients with COPD (81 ng/ml, p < 0.001) compared to healthy subjects (40 ng/ml). Patients with high plasma YKL-40 had a hazard ratio (HR) of 1.42 (95% CI: 1.15–1.75, p = 0.001) for all-cause mortality. Multivariate analysis showed that YKL-40 (HR 1.38; 95% CI: 1.11–1.72, p = 0.004), age (HR 1.05; 95% CI: 1.03–1.06, p < 0.0001), Severe COPD (HR 1.35; 95 CI: 1.03-1.76, p = 0.03) very severe COPD (HR 2.19; 95% CI: 1.60 - 2.99 < 0.0001), neutrophil granulocyte count (HR 1.05; 95% CI: 1.01-1.08, p = 0.01), and a smoking history of > 40 years (HR 1.38; 95% CI: 1.11-1.71, p = 0.003) were independent prognostic markers of OS.

**Conclusion:**

High plasmaYKL-40 is associated with increased mortality in patients with moderate to very severe COPD, suggesting a role for YKL-40 as a potential biomarker of mortality in this patient group.

**Trial registration:**

ClinicalTrials.gov: NCT00132860.

## Background

Airflow limitation is a central feature of chronic obstructive pulmonary disease (COPD). The airflow limitation is irreversible, and it is recognized that localized tissue destruction in response to inflammatory processes in lung tissue due to prolonged exposure to noxious gases like tobacco smoke is associated with the development of COPD (http://www.goldcopd.org – accessed 1 February, 2013). The disease is usually progressive, and it is one of the leading causes of death in the Western world [1,2]. In addition to localized inflammation in lung tissue, systemic low grade inflammation is recognized as part of the disease spectrum in COPD [3,4]. Basal levels of systemic inflammation could reflect disease activity and thus be a valuable tool in determining disease activity in patients with COPD.

The plasma concentration of YKL-40 (also called chitinase3-like-1 (CHI3L1)) has attracted attention as a biomarker of disease activity in a wide array of diseases hallmarked by chronic low grade inflammation, tissue remodeling, and fibrosis, e.g. cardiovascular diseases [5-7], asthma [8], diabetes mellitus type 1 [9] and 2 [10,11], rheumatoid arthritis [12], liver fibrosis [13-15], and cancer [16]. Furthermore, YKL-40 levels have been shown to be a strong predictor of overall mortality in patients admitted to hospital irrespective of diagnosis [17].

The crystal structure of YKL-40 is known [18]. YKL-40 is mainly secreted by cancer cells, macrophages, and neutrophils [16,19]. Studies suggest that YKL-40 plays a role in cell proliferation and differentiation [20], inflammation [21,22], extracellular tissue remodeling [21], and protection against apoptosis [23]. YKL-40 also induces cancer angiogenesis both independently and through stimulation of vascular endothelial growth factor [24]. In *Streptococcus pneumoniae* infected CHI3L1 null mice, YKL-40 is a regulator of antibacterial responses that augment antimicrobial resistance by contributing to bacterial killing and controlling bacterial dissemination [25].

Recently, it has also become increasingly evident that YKL-40 plays a role in inflammatory lung diseases. Increased concentrations of YKL-40 in plasma and bronchoalveolar lavage fluid are found in patients with asthma [8], COPD [26], and idiopathic pulmonary fibrosis (IPF) [27]. Interestingly, high YKL-40 levels predicted short survival in 85 patients with IPF [27]. When exposed to YKL-40, macrophages from COPD patients produce elevated levels of the pro-inflammatory biomarkers IL-8, MCP-1, MIP-1α, and MMP-9 [26], and YKL-40 is secreted from alveolar macrophages when these are stimulated by TNF-α. Serum YKL-40 is positively correlated to low-attenuation area percentage a marker of the extent of lung emphysema, and negatively correlated to forced expiratory volume in 1 second (FEV_1_)% predicted, a marker of disease severity, in patients with COPD [28] and patients with asthma [8]. A single nucleotide polymorphism in the promoter of the CHI3L1 gene (-131 C → G) of patients with asthma was correlated with elevated serum YKL-40, bronchial hyper reactivity, and pulmonary function [29]. Knockdown of the CHI3L1 gene in a human airway epithelia cell line protected the cell line against hypoxic cell damage [30], further substantiating the pro-inflammatory role of YKL-40 in inflammatory pulmonary disease.

In this study we investigated whether plasmaYKL-40 levels above the age-corrected 75% percentile were associated with long-term mortality in a group of patients with moderate to very severe COPD. We also examined whether there was a relationship between COPD severity and plasmaYKL-40 as previously reported. The hypothesis was that plasma concentrations of YKL-40 above the 75% age-corrected percentile reflect increased basal inflammation in patients with moderate to very severe COPD which is implied by an increased mortality rate in patients with COPD. We tested this hypothesis in 493 patients with COPD followed for 10 years.

## Methods

### Study population

In all, 575 patients with COPD were enrolled from May 2001 to April 2004 in a randomized clinical trial studying the effect of azithromycin 500 mg, 3 days per month for 36 months. Primary outcome was change in post-bronchodilator FEV_1_. Secondary outcomes included number of hospital admissions, number of days in hospital, mortality, quality of life, use of medication, prevalence of respiratory pathogens, and prevalence of macrolide resistance. Inclusion and exclusion criteria are explained in detail in Table 1, and the trial was registered at http://clinicaltrials.gov/- identifier NCT00132860 (accessed 1 September 2012). Ethical permission for the study was obtained from the Regional Scientific Ethical Committee for Southern Denmark, approval number VF 19990031. Written informed consent for participation in the study was obtained from all participants before inclusion.

**Table 1:** Inclusion and exclusion criteria for the study

**Inclusion criteria**	**Exclusion criteria**
• Patients above 50 years of age, with a prior admission for exacerbation of COPD within the last two years.	• Patients with end-stage COPD, who are not expected to survive for 3 years (typically bedridden patients being dyspnoeic at rest).
• Current or ex-smoker	• Patients with known other respiratory tract infection, e.g. tuberculosis or aspergillosis, in whom the intervention is known to be inefficient.
• Postbronchodilator FEV1 < 60% in stable condition (> 4 weeks after hospitalization)	• Patients with pulmonary malignancy
• < 300 ml bronchodilator reversibility in FEV1	• Patients with other pulmonary diseases than COPD
	• Patients with immunodeficiency. However, COPD patients treated with steroids can be included
	• Patients with known hereditary disposition to lung infections such as alfa-1-antitrypsin deficiency, cystic fibrosis or primary ciliary dyskinesia.
	• Patients receiving long-term antibiotic treatment
	• (e.g. recurrent cystitis)
	• Patients with known allergy or intolerance to azithromycin
	• Pregnant or breastfeeding women
	• Manifest heart, liver or renal insufficiency
	• Patients that, for reasons not stated above, are unlikely to be able to participate in a study period of 3 years.

### YKL-40 analysis and reference interval

Plasma samples for YKL-40 analysis were available from 493 patients. Bloodsampling was done at baseline a time where patients were in a stable phase of the disease i.e. no prior admissions within the last month and no antibitiotics used within the week leading up to blood sampling. Blood for EDTA plasma was centrifuged within 1 hour after blood sampling and then stored at -80°C until analysis. The plasma levels were analyzed in February 2011. YKL-40 concentrations in plasma has been shown to be stable for up to 16 years when frozen at -80°C degrees [31]. The plasma concentration of YKL-40 was determined in duplicate by a commercial enzyme-linked immunosorbent assay (ELISA) (Quidel, Santa Clara, CA, USA) according to the manufacturer’s instructions. The detection limit was 10 ng/ml, and intra- and inter-assay coefficients of variation (CVs) were < 5% and < 6%.

The reference interval for plasma YKL-40 was determined from a previous study in which 3130 healthy subjects (1837 women, 1293 men) aged 21–84 years from the Danish general population, the Copenhagen City Heart Study were examined for YKL-40 concentrations [31]. They had no known disease at the time of blood sampling in 1991–1994 and remained healthy and alive during the 16-year follow-up period. From this study an age dependent correlation was found between age and plasma concentrations of YKL-40 and a formula has been extrapolated from this study [31] which we applied to our present study.

### Statistics

For all participants, person-years of follow-up were computed from their inclusion in the study (May 2001 to April 2004) until date of death, emigration, lost to follow-up, or 31 January 2011 (last day of follow-up), whichever came first. Patients’ date of death was registered in the Danish Central Registry. Follow-up at 10 years was 99.4% complete.

Primary endpoint was overall survival (OS). Analyses of measurements for time to death were done using the Cox proportional hazards model. Patients were divided into two groups according to plasma YKL-40: concentrations higher than the 75th percentile for age-matched healthy subjects (high levels) and normal levels. This was performed using an equation from a previous report of plasma YKL-40 in 3130 healthy subjects in which the 75th percentile was used as a cut-off value to define a high YKL-40 level [31]. Survival probabilities for OS were estimated by the Kaplan-Meier method, and tests for differences between strata were done using the log-rank statistic.

Multivariate analysis included plasmaYKL-40 above 75% of the age-adjusted level, COPD stage as defined by the GOLD initiative i.e. Moderate COPD ( 79–50 FEV1% predicted), severe COPD (30–50 FEV1% predicted) [32], and very severe COPD (< 30 FEV1% predicted), Charlson Comorbidity Index > 2, age (as a continuous variable), treatment group (azithromycin vs. placebo), and gender as possible confounders. In addition to these, potential confounders were tested in univariate analysis and included in the final model if they were significant at a level of 0.25 or below. The number of events (376 patients died) was within the 10 events per variable suggested by Peduzzi et al. [33].

Variables were tested for interaction by likelihood ratio test statistics and no significant interactions were found. Continual confounders were all tested for linearity. Proportional hazards assumptions were tested individually for each confounder. We used log-log plots and observed vs. expected plots for categorical confounders and observed vs. expected plots for continuous confounders. No violation was displayed on confounders. Further analysis was performed using multivariate Cox proportional hazards models. Results were presented as median with 95% confidence interval (CI) or interquartile range or rates as appropriate. All statistical analyses were carried out using STATA 11.1 (Stata Corp LP, TX, USA).

## Results

### Study population characteristics

The study population consisted of 493 individuals (247 males and 246 females) characterized by fairly advanced COPD, with a median FEV_1_% predicted of 38.5%. Of these, 129 (26%) had moderate COPD, 250 (51%) had severe, and 114 (23%) had very severe COPD. Median plasma YKL-40 was increased in patients with COPD (81 ng/ml, range 13–925 ng/ml) compared to healthy controls 40 ng/ml). In all, 376 (76%) patients died during the 10-year follow-up period.

Patients were divided into two groups defined by plasma YKL-40: higher (n = 171) or below (n = 322) the age-corrected 75% percentile of plasma YKL-40 in a large group of healthy subjects. Table 2 gives clinical characteristics of the patients. The groups displayed a homogenous composition, but plasmaYKL-40 above the 75% age-corrected percentile was associated with a higher Charlson Score Index.

**Table 2:** Baseline variables distributed according to plasma concentrations of YKL-40

	**< 75% percentile (n = 322)**	**> 75% percentile (n = 171)**	**p-value**
**Age at index date‡**	70 (65–75)	72 (66–76)	0.16
Charlson score‡*	1 (1–2)	1 (1–3)	0.02
**Smoking (years)‡**	37 (26–50)	40 (28–55)	0.13
**Neutrophils (x10^9/L)‡**	6.2 (4.7-8.0)	6.3 (4.9-8.9)	0.18
**FVC (L)‡**	2.00	1.91	0.09
**FEV**_**1 **_**(L)‡**	0.89 (0.69-1.15)	0.91 (0.69-1.13)	0.72
**FEV1 predicted (%)‡**	37.69 (29.73-47.25)	40.68 (31.03-49.67)	0.23
**BMI‡**	24.20 (21.04-27.83)	24.01 (20.08-27.24)	0.20
**Present smokers§ (%)**	120 (37)	76 (44)	0.12
**Male gender†§ (%)**	164 (51)	83 (49)	0.61
**Randomization azithromycin§ (%)**	164 (51)	84 (49)	0.70

*Significant difference using Kruskal-Wallis equality-of-populations rank test.

‡Values are median (interquartile range).

§Values are number (%).

### Plasma YKL-40 and FEV_1_

**Figure 1:**
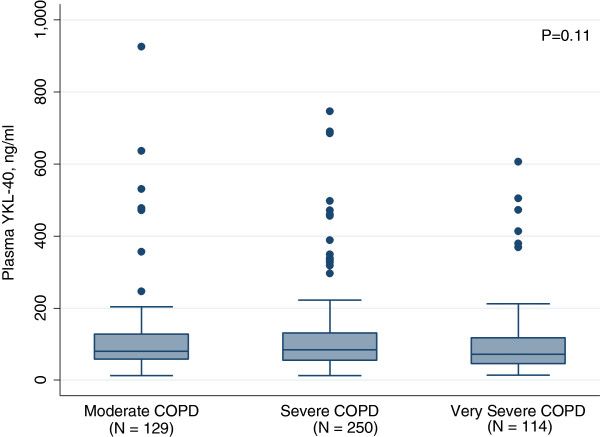
**Plasma YKL-40 and COPD severity.** Boxplots of plasma concentrations of YKL-40 in patients with COPD according to disease severity. Moderate COPD (79–50 FEV1 % predicted), severe COPD (30–50 FEV1 % predicted) and very severe COPD (< 30 FEV1 % predicted). The median score is the line in the middle of the box, and the 25^th^ and 75^th^ percentiles are the lower and upper part of the box. The whiskers are the 5^th^ and 95^th^ percentiles. Outliers are given as dots. No significant differences were found (Kruskal-Wallis test).

We also investigated whether there was an association between plasma YKL-40 and lung function. The results are displayed in the subsection “Plasma YKL-40 and COPD severity” (Figure 1).

### Univariate survival analysis

Univariate analysis showed that plasma YKL-40 dichotomized to levels above the age-adjusted 75% percentile in healthy subjects was associated with shorter OS (HR = 1.42, p = 0.001) (Table 3). In addition to plasma YKL-40 levels, age, neutrophil granulocyte count, severe/very severe COPD, and a smoking history of more than 40 pack years were also associated with increased mortality (Table 3).

**Table 3:** Univariate analysis of potential predictors of mortality

	**Hazard rate**	**95% Confidence interval**	**p-value**
High vs. normal plasma YKL-40**	1.42	1.15 – 1.75	0.001
Age*	1.04	1.03 – 1.06	<0.001
Neutrophils†	1.05	1.01 – 1.09	0.006
Charlson score index > 2	1.21	0.94 – 1.54	0.14
Moderate COPD‡	Ref. value	Ref. value	-
Severe COPD‡	1.38	1.06 – 1.77	0.014
Very severe COPD‡	1.94	1.45 – 2.60	<0.001
Smoking at baseline	1.10	0.90 – 1.35	0.36
BMI < 20	1.17	0.91 – 1.50	0.21
Pack years > 40	1.29	1.05 – 1.58	0.014

*Estimated hazard ratio associated with an increment of one year.

**Patients were dichotomized according to the 75th percentile of plasma YKL-40 in age-matched healthy subjects (30).

†Estimated hazard ratio associated with an increment of 1 * 10^9^ cells/L.

‡Moderate COPD (79–50 FEV1 % predicted), severe COPD (30–50 FEV1 % predicted) [32], and very severe COPD (< 30 FEV1 % predicted).

**Figure 2:**
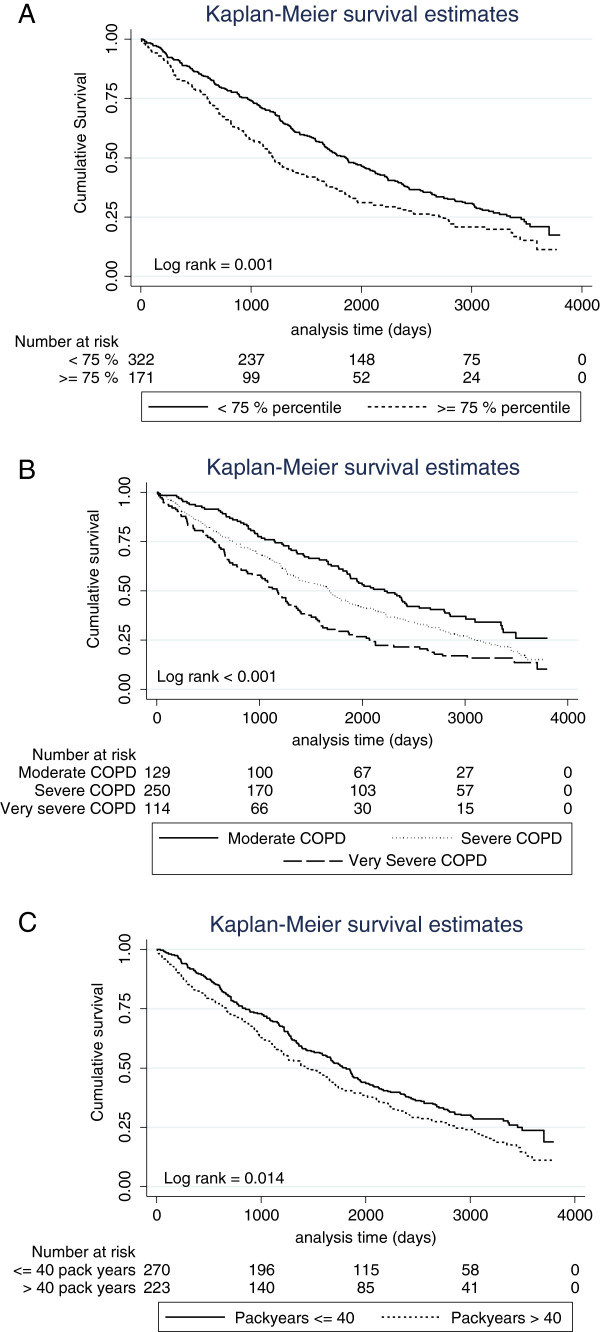
**Kaplan-Meier survival curves showing the association between plasma YKL-40 and 10-year OS.** Patients were dichotomized according to 75^th^ percentile of plasma YKL-40 in age-matched healthy subjects **(A)**, COPD severity **(B)**, and 40 pack years **(C)**. P-value refers to the log-rank test for equality of strata. Moderate COPD (79–50 FEV1 % predicted), severe COPD (30–50 FEV1 % predicted) and very severe COPD (< 30 FEV1 % predicted).

**Figure 3:**
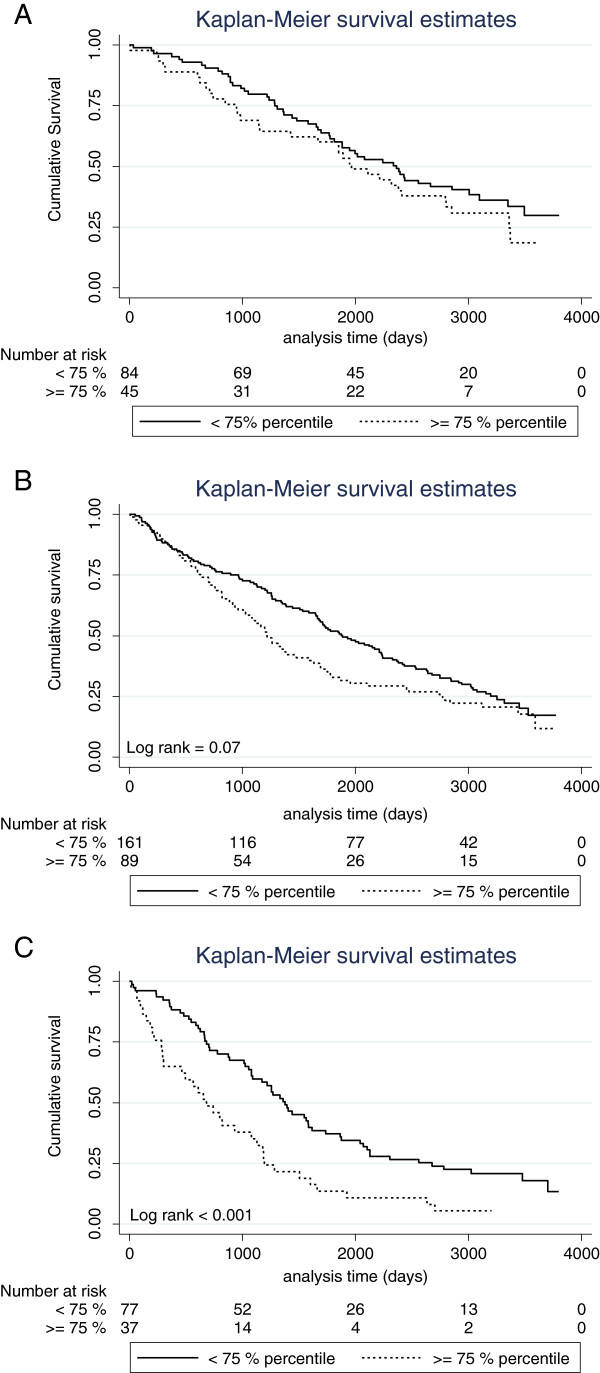
**Kaplan-Meier survival curves showing the association between plasma YKL-40 and 10 year OS in patients with different degree of disease severity.** Patients were dichotomized according to the 75^th^ percentile of plasma YKL-40 in age-matched healthy subjects. Patients were divided into groups with moderate COPD **(A)**, severe **(B)** and very severe COPD **(C)**. P-value refers to the log-rank test for equality of strata.

Figure 2 gives Kaplan-Meier curves for COPD patients according to different categorical variables associated with OS in the univariate analysis (2A: high YKL-40 vs. normal; 2B: COPD severity; 2C: pack years above 40 vs. lower levels). Patients with high plasma YKL-40 had a 50% cumulative survival of only 40 months in contrast to patients with normal plasma YKL-40 who had a 50% cumulative survival of 62 months. We also investigated whether high levels of plasma YKL-40 retained a discriminative effect when patients were stratified into different levels of COPD severity. Even though this was not the case for patients with moderate COPD, such an association was displayed for patients with severe and very severe COPD (Figure 3A-C).

### Multivariate survival analysis

To determine the independent effect of plasma YKL-40 on OS, we included age and neutrophil granulocytes as continuous variables and Charlson Comorbidity Index, COPD severity, and pack years as categorical variables. Patients with COPD and high age-adjusted plasma YKL-40 had shorter OS (HR = 1.39, 95% CI: 1.12–1.73, p = 0.004). Age (HR = 1.04, p < 0.0001), neutrophil granulocyte count (HR = 1.04, p = 0.03), severe COPD (HR = 1.33, p = 0.04), very severe COPD (HR = 2.22, p < 0.0001), and a smoking history in excess of 40 pack years (HR = 1.36, p = 0.009) were also independent parameters of short OS (Table 4).

**Table 4:** Multivariate proportional hazards Cox regression of prognostic markers for mortality

	**Adjusted hazard ratio**	**95% Confidence interval**	**p-value**
High vs. normal plasma YKL-40**	1.38	1.11 - 1.72	0.004
Age*	1.05	1.03 - 1.06	<0.0001
Neutrophils†	1.05	1.01- 1.08	0.01
Moderate COPD‡	Ref. Value	Ref. Value	Ref. Value
Severe COPD‡	1.35	1.03 - 1.76	0.03
Very severe COPD‡	2.19	1.60 - 2.99	<0.0001
Pack years > 40	1.38	1.11- 1.71	0.003

*Estimated hazard ratio associated with an increment of one year.

**Patients were dichotomized according to the 75th percentile of plasma YKL-40 in age-matched healthy subjects (30).

†Estimated hazard ratio associated with an increment of 1 * 10^9^ cells/L.

‡Moderate COPD (79–50 FEV1 % predicted), Severe COPD (30–50 FEV1 % predicted) [32].

and Very Severe COPD (< 30 FEV1 % predicted).

## Discussion

COPD is characterized by a localized and usually progressive destruction of lung tissue, and increasing awareness has been given to the systemic effects of COPD. It is believed that the ongoing inflammation in the lungs “overspills” into the systemic circulation. Monitoring of systemic inflammatory biomarkers may reflect disease activity in patients with COPD, and would help to monitor disease progression, treatment efficacy, and identification of COPD phenotypes that would benefit from disease modifying pharmacotherapy.

Several putative inflammatory biomarkers in plasma or serum like C-reactive protein (CRP) [34,35], pulmonary and activation-regulation chemokine (PARC/CCL-18) [36], and fibrinogen [37] have been tested for their ability to predict all-cause and COPD-related mortality in patients with various stages of COPD. Despite showing promise as prognostic biomarkers, serum CRP and fibrinogen are not modified by potent inflammation modifying medications [38]. A general consensus on the ability of serum CRP to predict mortality was challenged in a study which was unable to demonstrate the same predictive value of serum CRP on mortality in patients with moderate to very advanced COPD [39]. Furthermore, the repeatability of serum CRP in patients with COPD and stable disease showed a high degree of variability, suggesting that the use of serum CRP as a biomarker of basal disease activity in patients with COPD is unfeasible [40].

The reasons for these ambiguous results can be many, but a recent study found that only a subset of patients with COPD is characterized by persistent systemic inflammation and the authors propose that a clinical phenotype with persistent inflammation is the reason for this [41]. In this study patients with persistently elevated levels of a select group of inflammatory biomarkers (IL-6, CRP, fibrinogen and white blood cells) were associated with an adverse outcome. These findings are very interesting as a very recent study found that IL-6 levels increased during a three year period whereas no change was apparent in mean CRP levels. IL-6 levels correlated with six-minute walk distance and mortality further corroborating a role of IL-6 as a marker of persistent inflammation [42] an interesting finding as a fairly recent study found that IL-6, but not TNF-α, stimulates YKL-40 production. These results suggest that IL-6 could be an upstream activator of YKL-40 independent of TNF-α [43]. In the present study of patients suffering from moderate to very severe COPD, we found that a high plasma concentration of YKL-40 was an independent predictor of shorter OS. This is a novel observation in COPD patients. The study benefited from a fairly large number of 493 well-characterized patients, and within the study period of 10 years, follow-up was almost complete (99.4% complete). In addition to this, the study population carried a very high fatality rate, and more than 76% of the population died during the study period.

Our primary outcome was all-cause mortality. We did not have access to cause-specific mortality in this study. It is well known that patients suffering from COPD are subject to co-morbidities, e.g. lung cancer and cardiovascular disease, which are associated with elevated plasma concentrations of YKL-40 and increased mortality [5,6,16,44]. We cannot rule out that these causes of death were a contributing factor to death in our cohort. However, the patients were excluded from inclusion into the study if they suffered from pulmonary malignancies, other pulmonary disease, or if they were suffering from advanced heart or kidney disease (Table 1). COPD as a primary cause of death is underreported, and hence a more general inquiry into causes of death would most likely underestimate COPD as cause of death [45].

Our study suffered from lack of a healthy control group. When we compared plasma YKL-40 of the patients with age-adjusted plasma YKL-40 in a large group of 3130 healthy subjects from the general population [31], we found that 35% of the patients with COPD had a plasma YKL-40 level higher than the age-corrected 75% level in healthy subjects. In the literature, the cut-off values for high/normal plasma YKL-40 in patients with cancer are often set at 90% or 95%. If these cut-offs were used, 17% of the COPD patients had a plasma YKL-40 level higher than the 90% upper normal level, and only 8% of the COPD patients had a plasma YKL-40 level higher than the 95% upper normal level. We initially decided to use the age-corrected 75% percentile because we deemed it reasonable that levels above this cut-off would indicate low- grade increased inflammatory activity in COPD. We thought it less likely that plasma YKL-40 would elevate to levels comparable to those seen in metastatic cancer.

Corticosteroids decrease plasma concentrations of YKL-40 [46]. The use of corticosteroids was not registered in our cohort of COPD patients at the time of enrollment. But the blood samples used for determination of YKL-40 were drawn at time of inclusion in the study when the patients were in a stable disease phase, and only a minor group of patients were probably treated with oral corticosteroids. Patients were excluded if they had been treated with antibiotics up to 1 week before inclusion or if they had a history of hospitalization within the last month. This should minimize the risk of patients having active infections or exacerbations present at the time of blood sampling and would give a more accurate picture of chronic inflammation in patients with COPD. We cannot exclude that we underestimated the plasma levels of YKL-40 in individual cases.

The original trial investigated the effects of the antibiotic azithromycin on a number of outcomes. Azithromycin is a potent antibiotic but is also an anti-inflammatory drug. This serves as a confounder when interpreting our results. The distribution of patients receiving azithromycin was comparable in patients with low or high plasma YKL-40 (Table 2). We were unable to demonstrate an effect of azithromycin on OS in the univariate model or the final multivariate model (results not shown). In addition to this, the final multivariate model was tested in a model in which we stratified into a group who had received treatment vs. one that had not. These groups were then examined using a likelihood ratio test in which we compared the two models against each other and no difference was found (results not shown). Thus we do not believe this potential confounder had any impact on the outcome of this study.

An association between mortality and increased plasma concentrations of YKL-40 does not prove causation. The finding is interesting, however, as the same observation has been made in patients suffering from IPF [27]. Increased levels of several key inflammatory mediators are secreted from macrophages in patients suffering from COPD, e.g. IL-8, MCP-1, MIP-1α and MMP-9 when stimulated by YKL-40 [26]. Intriguingly, stimulation of macrophages with TNF-α results in increased secretion of YKL-40 [26]. This potentially places YKL-40 centrally in the inflammatory cascade between upstream signaling through TNF-α and downstream signaling via IL-8, MCP-1, MIP-1α, and MMP-9. These findings were recently contested however and the role of YKL-40 in airway inflammation remains to be elucidated (see above) [43].

The notion that high plasma YKL-40 is associated with increased inflammatory response and a rapid decline of lung function is supported by our findings of short OS. Paraclinical findings in another study support this hypothesis as high plasma YKL-40 was associated with high levels of low-attenuation area percentage and a negative correlation to FEV_1_% predicted [28].

Other potential markers of short OS were tested in the present study. As expected, patients with severe or very severe COPD had shorter OS than did patients with moderate COPD. We could not confirm that BMI and Charlson Comorbidity Index were prognostic parameters. The median BMI of 24 in our cohort was high, and only 100 (20%) patients had BMI < 20. Twenty-six percent of our patients had moderate COPD, and Charlson Comorbidity Index may not accurately predict mortality in patients suffering from advanced COPD. Such patients are already at an increased risk of dying, and co-morbidities may play a lesser role.

No association was found between FEV_1_% predicted and plasma YKL-40 in our study. This is in contrast to two earlier small studies of 45 and 50 patients with COPD [26,28] reporting that plasma YKL-40 was related to disease stage, i.e. related to the degree of airway obstruction measured by FEV_1_% predicted. The patients in these studies suffered from a lesser degree of obstruction and were younger than our patients, which may account for some of the difference.

The parameter FEV_1_% predicted represents disease staging and does not provide information about how fast the disease developed to the current level of airway obstruction, an observation that corresponds well with the recent changes in the GOLD COPD disease staging guidelines (http://www.goldcopd.org – accessed 17 April, 2013) in which symptoms and exacerbations also have an influence on disease staging. This may explain why we see this discrepancy in our cohort. YKL-40 may more accurately describe disease activity, whereas FEV_1_% represents the current level of lung damage. This notion is supported by our findings: a high plasma YKL-40 in patients with severe and very severe COPD predicted a worse outcome independent of the traditional disease staging levels (Figure 3B-C). This could potentially signify that these patients had a disease characterized by a higher degree of inflammation and that plasma YKL-40 is able to assign patients with advanced disease to a high- and a low-risk group independent of disease severity.

Identification of biomarkers that can predict progression of COPD remains a high priority. Currently we only have an indirect measure of COPD disease progression through the use of spirometry. A biomarker level in a blood sample able to assess the inflammatory activity in patients with COPD would prove a valuable tool in monitoring disease activity, treatment efficacy, and prognosis of patients. This could help to identify patients characterized by high inflammatory activity who might benefit most from inflammatory modifying therapies.

## Conclusion

In conclusion, our study supports the hypothesis that plasma YKL-40 is elevated in many patients suffering from advanced COPD. Patients with the highest plasma YKL-40 had the shortest OS. The exact functions of YKL-40 in disease progression of COPD remain unknown. Prospective, longitudinal studies of patients with COPD are needed in which plasma YKL-40 is determined several times during follow-up and related to clinical characteristics, e.g. loss of FEV_1_. It would also be interesting to investigate whether high plasma YKL-40 is associated with an increased susceptibility to exacerbations of COPD. Increased inflammatory activity could potentially lead to exaggerated responses to inflammatory insults to the airways, and plasma YKL-40 may be a new prognostic biomarker in patients with COPD.

## Competing interests

The study was supported by grants from the Research Council of Southern Denmark, Overlægerådets Legatråd, the Danish Lung Association, and Danish Council for Independent Research (grant no. 22-04-0636). The study sponsors had no role in the design and conduct of the study; in the collection, management, analysis, and interpretation of the data; or in the preparation, review, or approval of the manuscript. The authors had full access to all the data in the study and had the final responsibility for the decision to submit the manuscript for publication.

## Authors’ contributions

All authors conceived and designed the study or analyzed the data; all authors contributed to and approved the final draft of the manuscript; LHM, CP, and SSP collected study data; JSJ conducted plasma YKL-40 analysis; DBH conducted statistical analyses.

## Pre-publication history

The pre-publication history for this paper can be accessed here:

http://www.biomedcentral.com/1471-2466/13/77/prepub
